# Interferon-Gamma Primed Human Clonal Mesenchymal Stromal Cell Sheets Exhibit Enhanced Immunosuppressive Function

**DOI:** 10.3390/cells11233738

**Published:** 2022-11-23

**Authors:** Celia M. Dunn, Sumako Kameishi, Yun-Kyoung Cho, Sun U. Song, David W. Grainger, Teruo Okano

**Affiliations:** 1 Cell Sheet Tissue Engineering Center (CSTEC), Department of Molecular Pharmaceutics, University of Utah, Salt Lake City, UT 84112, USA; 2 Department of Biomedical Engineering, University of Utah, Salt Lake City, UT 84112, USA; 3 SCM Lifescience Co., Ltd., Incheon 21999, Republic of Korea; 4 Institute for Advanced Biomedical Sciences, Tokyo Women’s Medical University, Tokyo 162-8666, Japan

**Keywords:** mesenchymal stem cells, immunomodulation, pre-conditioning, licensing, tissue engineering, cellular therapy, coculture

## Abstract

Mesenchymal stromal cells (MSCs) represent a promising treatment for immune-related diseases due to their diverse immunomodulatory paracrine functions. However, progress of culture-expanded MSCs is hindered by inconsistent cell function, poor localization, and insufficient retention when administered as suspended cell injections, thus placing spatiotemporal dosing constraints on therapeutic functions. To address these limitations, we introduce the combination of in vitro interferon-gamma (IFN-γ) priming, a key stimulator of MSC immunosuppressive potency, and thermoresponsive cultureware to harvest cultured MSCs as directly transplantable scaffold-free immunosuppressive cell sheets. Here, we demonstrate that MSC sheets produced with IFN-γ priming upregulate expression of immunosuppressive factors indoleamine 2,3-dioxygenase (IDO-1), interleukin-10 (IL-10), programmed death ligand-1 (PD-L1), and prostaglandin E2 (PGE2) in both dose- and duration-dependent manners. In addition, IFN-γ primed MSC sheets showed increased ability to inhibit T-cell proliferation via indirect and direct contact, specifically related to increased IDO-1 and PGE2 concentrations. Furthermore, this study’s use of human clinical-grade single-cell-derived clonal bone marrow-derived MSCs, contributes to the future translatability and clinical relevancy of the produced sheets. Ultimately, these results present the combination of IFN-γ priming and MSC sheets as a new strategy to improve MSC-mediated treatment of localized inflammatory diseases.

## 1. Introduction

Mesenchymal stromal cells, also called mesenchymal stem cells (MSCs), have long been advocated as a cell therapy for immune-related diseases due to their ability to dynamically respond to and attenuate inflammation through their secretion of anti-inflammatory factors [1,2]. However, >900 MSC clinical trials to date exhibit inconsistent results [3], and only three MSC therapies are so far approved for immune-related diseases globally (none yet approved in USA) [4]. Clinical administration of MSCs has repeatedly demonstrated safety but has generally failed so far to corroborate therapeutic effects observed in preclinical in vitro and in vivo models [5,6]. This provides new opportunities to re-think current standard practices utilizing suboptimal injection-based delivery of “naïve” (i.e., heterogeneous, non-primed, non-licensed, non-standardized) MSC suspensions [7,8,9]. New MSC products must consider and address the challenges associated with standards for MSC potency and dosing, including route of administration and pharmacological disposition of MSCs prior to delivery [3,9,10].

MSC-mediated immunosuppression is a multifaceted process involving paracrine signaling via secreted soluble factors and transmembrane surface proteins such as interleukin 10 (IL-10) [11,12], prostaglandin E2 (PGE2) [13,14], indoleamine 2,3-dioxygenase (IDO-1) [15,16,17,18], programmed death ligand 1 (PD-L1) [19,20], and human leukocyte antigen G (HLA-G) [21]. However, at baseline, “naïve” MSCs exhibit minimal immunosuppressive factor expression and activity, which become upregulated in response to instructive inflammatory milieu factors (i.e., IFN-γ, TNF-α, IL-17) [17,22]. In the presence of pro-inflammatory factors (e.g., IFN-γ, TNF-α, IL-17), MSCs are widely reported to activate regulatory T-cells [23], inhibit effector T-cell activation [12,17,19,22,23], inhibit B-cell proliferation and differentiation [24,25], and induce M2 macrophage polarization [17]. Delivery of “naïve” MSCs confounds therapeutic results by relying on activation by the patients’ own inflammatory cues in vivo, namely IFN-γ [26], and is posited to be a primary contributor to heterogeneity in patient responses [27,28,29]. Priming (i.e., pre-treatment, licensing) MSCs with pro-inflammatory factors before delivery is proposed to overcome this barrier to achieve improved, more consistent therapeutic responses [30]. IFN-γ has been shown to be an essential activator of MSC-mediated immunosuppression [27,31,32] by upregulating MSC production of IDO-1 [17]. Preclinical studies have reported improved efficacy of IFN-γ primed MSCs compared to naïve, non-primed, MSCs in animal models of graft-versus-host-disease [26], colitis [33], and renal fibrosis [34]. However, injected cell suspensions of IFN-γ primed MSCs remain hindered by poor cell localization and retention at the disease site [35], diminishing sustained MSC-derived immunomodulatory factor doses and drastically increasing the number of cells needed to treat humans [27]. To this end, cell sheets are an attractive strategy to address the limitations of injected cell suspensions by improving localized MSC engraftment and retention.

Cell sheets are a scaffold-free, directly transplantable alternative to conventional injection-based delivery of MSC suspensions [36]. Cell sheet tissue engineering uses thermo-responsive cell cultureware to harvest cultured cells as readily transplantable three-dimensional (3D) cell sheet constructs retaining cell adhesive extracellular matrix. This enables superior engraftment and localization compared to injected cell suspensions in several injury models [37,38,39,40]. The long-term retention of directly transplanted MSC sheets has demonstrated enhanced therapeutic efficacy in preclinical rat models by improving sustained MSC production of pro-regenerative paracrine factors HGF and VEGF [39,41]. Hence, transplanted, highly immunomodulatory MSC sheets are promising to advance MSC-mediated treatment of immune-related disorders, by facilitating sustained production of immunosuppressive paracrine factors. However, the immunosuppressive ability of allogeneic MSCs as 3D cell sheets remains underreported and requires further investigation to establish an immunosuppressive hcBMSC sheet for future clinical use.

For this study, we used human clonal (single-cell-colony-derived) bone marrow MSCs (hcBMSCs) [42], which represent a more homogenous MSC population important to future clinical translation. Conventional BMSC isolation methods result in functionally heterogeneous MSC populations comprising unique subset populations with variable immunomodulatory/regenerative potentials [43,44,45,46]. Throughout passage expansion, the composition of MSC subset populations changes [47], complicating the ability to reliably expand and bank consistent MSCs for clinical use. Automated label-free technologies are emerging to facilitate improved MSC purification and selection using select properties [48,49,50,51,52]. Single-cell derived clonal MSCs exhibit long-term cell proliferation at late passage numbers (P10) with low immunogenicity and high regenerative capacity [53,54,55]. Therefore, to increase the clinical translatability of our study, we use clinical-grade single-cell originating hcBMSCs with demonstrated clinical safety and efficacy in early phase clinical trials [28,56]. This study aimed to combine hcBMSCs and scaffold-free cell sheet technology with IFN-γ priming to design a highly immunosuppressive new MSC sheet local therapy for future clinical translation. We hypothesized that IFN-γ priming of MSC sheets would produce a highly immunosuppressive, transferable construct. To functionally assess the immunosuppressive potential of “naïve” (non-primed) and IFN-γ primed MSC sheets, we used both in vitro direct and indirect (transwell) cocultures with human peripheral blood mononuclear cells (hPBMCs) to investigate the suppression of T cell proliferation.

## 2. Materials and Methods

### 2.1. hcBMSC Culture

hcBMSCs (clonal line: A106 D127) were received from SCM Lifesciences (Incheon, Republic of Korea) at passage 6 and were expanded to generate a passage 8 working cell bank. hcBMSCs supplied by SCM Lifesciences were produced from human bone marrow aspirates by isolating single-cell-derived clonal BMSCs using their patented Subfractionation Culturing Method [42]. hcBMSCs were confirmed positive for MSC markers CD73, CD105, and CD90, negative for CD45, CD34, and HLA-DR (Biolegend, San Diego, CA, USA), and expressed trilineage differentiation capabilities. hcBMSCs were plated at 1500–2000 cells/cm^2^ in growth media containing Low Glucose (1 g/L) Dulbecco’s Modified Eagle’s Medium (LG-DMEM) (Gibco, Waltham, MA, USA) supplemented with 10% fetal bovine serum (FBS) (Thermo Fisher Scientific, Cambridge, MA, USA), 1% penicillin-streptomycin (PS) (Gibco, Waltham, MA, USA), and 0.5 µL/mL MycoZap Prophylactic (Lonza, Basel, Switzerland) and incubated in a humidified environment (37 °C, 5% CO_2_). Media was changed after 24 h of initiating culture and every 2 days subsequently.

### 2.2. hcBMSC Cell Sheet Fabrication

Passage 9 hcBMSCs were collected from cell culture flask using 0.05% Trypsin–EDTA (Gibco, Waltham, MA, USA), and the cell viability and count was determined by trypan blue staining (Sigma-Aldrich, St. Louis, MO, USA) on a hemocytometer. The resultant passage 10 hcBMSCs were seeded onto 35 mm temperature-responsive UpCell^TM^ dishes (TRCDs) (Cellseed, Tokyo, Japan) at 4 × 10^5^ (~41,666 cells/cm^2^) per cell sheet in complete growth media supplemented with 50 μg/mL l-ascorbic acid 2-phosphate (Wako, Osaka, Japan) and allowed to culture for 6 days, with one media change on day 4. To prime the cell sheets, 25 ng/mL IFN-γ (Sigma-Aldrich, St. Louis, MO, USA) was added either at day 0, 2, or 4 of the 6-day cell sheet culture period and refreshed during the media change on day 4. On day 6, cells were moved to room temperature for 20 min, then detached with forceps as 3D cell sheets. Top-down microscopic images of each group prior to detachment were obtained using phase-contrast microscopy (AX10 microscope, Carl Zeiss Microimaging, Göttingen, Germany). Top-down macroscopic images of the resultant cell sheets were obtained immediately following detachment (0 h, immediately following detachment). To determine cell sheet viability, cell sheets were dissociated to produce a cell suspension via collagenase (Sigma Aldrich, St. Louis, MO, USA) and 0.25% Trypsin-EDTA (Gibco, Waltham, MA, USA) treatment, followed by trypan blue staining (Sigma Aldrich, St. Louis, MO, USA).

### 2.3. Histological Analysis

Immediately following detachment, cell sheets were fixed with 4% paraformaldehyde (Thermo Scientific, Waltham, MA, USA) for 20 min and paraffin-embedded (ARUP Laboratories, Salt Lake City, UT, USA). Embedded samples were sectioned at a 4 µm thickness. To visualize cell sheet structure, Hematoxylin (Sigma-Aldrich, St. Louis, MO, USA) and Eosin (Thermo Scientific, Waltham, MA, USA) (H&E) staining was conducted. Stained sections were imaged with a BX 41 widefield microscope (Olympus, Tokyo, Japan) using AmScope Software (v4.8.15934, USA) and AmScope 18MP Aptina Color CMOS camera (AmScope, Irvine, CA, USA).

### 2.4. hcBMSC Sheet—Hpbmc Coculture Assays

A proliferative T cell coculture assay was used to measure the immunosuppressive activity of hcBMSC sheets. Human peripheral blood mononuclear cells (hPBMCs) were isolated from a whole buffy coat from a healthy de-identified donor supplied by The Blood Center (New Orleans, LA, USA) using Histopaque-1077 (Sigma-Aldrich, St. Louis, MO, USA) based on ficoll density gradient centrifugation following the product protocol and washed three times in 1× PBS. HPBMCs were cryo-banked at passage 0 in 10% DMSO in FBS. Hpbmc seeding density was fixed at 1 × 10^6^ cells per hcBMSC sheet to establish 1:2.5 hcBMSC: Hpbmc (based on initial 4 × 10^5^ MSC sheet seeding). Detached IFN-γ primed hcBMSC sheets were transferred to the bottom of 24-well plates and allowed to attach for at least 1 h at 37 °C in minimal cell culture media. While the cell sheets were attaching, cryopreserved hPBMCs were thawed in Roswell Park Memorial Institute (RPMI) 1640 (Gibco, Waltham, MA, USA) supplemented with 10% FBS and 1% penicillin-streptomycin (Gibco, Waltham, MA, USA) and stained with CFSE dye (Cayman Chemical, Ann Arbor, MI, USA) according to manufacturer’s protocols to investigate cell proliferation [52]. Following CFSE-staining and neutralization, hPBMCs were activated by anti-CD3/anti-CD28 Dynabeads (ThermoFisher, Waltham, MA, USA), per protocol, to stimulate cell proliferation and either seeded onto a 0.4 µm-diameter pore transwell insert (Corning, Corning, NY, USA) placed over the attached hcBMSC sheets (indirect coculture) or directly onto the hcBMSC sheets (direct coculture) in complete RPMI 1640 media (Gibco, Waltham, MA, USA) supplemented with 30 U/mL Ril-2 (STEMCELL Technologies, Vancouver, Canada). Independently cultured (in the absence of cell sheet) (−)CFSE hPBMCs without Dynabead stimulation and (+)CFSE hPBMCs with and without Dynabead stimulation were used as positive and negative controls for flow cytometry analysis. After 4 days of culture, the hPBMCs were collected and the proliferative population was evaluated by measuring CFSE staining using a BD Canto cytometer. Anti-CD3, anti-CD4, and anti-CD8 (Biolegend, San Diego, CA, USA) were used to confirm the T-cell population in cultured hPBMCs (Supplemental Figure S1). Percent proliferation and proliferation index were quantified using FlowJo analysis software. Percent proliferation of T cells was reported as the percent of T cells in coculture that entered at least one cell division, normalized to the percent proliferation of the T cells cultured independently (control group). Proliferation index was quantified using the proliferation modeling function in the FlowJo analysis software and normalized to the proliferation index of the T cells cultured independently (control group).

### 2.5. Quantitative Real-Time PCR Analysis

Total RNA was isolated from hcBMSC sheets at day 0, immediately following detachment using the Qiagen Rneasy Mini Kit (Qiagen, Hilden, Germany) according to manufacturer instructions. Isolated tot RNA was quantified with a NanoDrop Spectrophotometer (Thermo Scientific, Waltham, MA, USA), and Cdna samples were prepared from 1.0 μg or 0.5 μg of total RNA/sample using a high-capacity Cdna Reverse Transcription Kit (Life Technologies, Carlsbad, CA, USA) following the protocol. Comparisons between samples were conducted on samples prepared with equal amount of total RNA per sample. Gene expression levels were quantified by real-time quantitative PCR (Qrt-PCR) performed on Applied Biosystems Step One Plus (Applied Biosystems, Waltham, MA, USA) using Taqman primers glyceraldehyde 3-phosphate dehydrogenase [GAPDH, Hs99999905_m1] as a housekeeping gene, interleukin-10 [Il10, Hs00961622_m1], indoleamine 2,3-dioxygenase [IDO1, Hs00984148_m1], prostaglandin E synthase 2 [PTGES2, Hs00228159_m1], major histocompatibility complex, class II, DR alpha [HLA-DRA, Hs00219575_m1], and CD274/programmed death-ligand 1 [CD274, Hs00204257_m1] (ThermoFisher Scientific, Waltham, MA, USA). Gene expression was normalized to GAPDH expression levels, and relative gene expression was determined using the comparative threshold cycle (CT) change algorithm normalized to non-primed (control) cell sheets.

### 2.6. Protein Secretion Assays

After 4 days of coculture, supernatants from sample and control groups were collected, centrifuged at 210× *g* for 5 min to remove suspended cells, and stored at −80 °C for ELISA. The concentration of soluble IL-10, IDO, PGE2, and PD-L1 per sample was quantified using human IL-10 DuoSet ELISA kit (R&D Systems, Minneapolis, MN, USA), human indoleamine 2,3-dioxygenase/IDO DuoSet ELISA kit (R&D Systems, Minneapolis, MN, USA), prostaglandin E2 Parameter Assay Kit (R&D Systems, Minneapolis, MN, USA), and human PD-L1 DuoSet ELISA Kit (R&D Systems, Minneapolis, MN, USA), respectively.

### 2.7. Statistical Analysis

For gene expression data statistical analysis was conducted on data sets of 2 separate frozen hcBMSC aliquots with 3 replicates per aliquot to total *n* ≥ 6 per experimental group. For coculture data, flow cytometry, and ELISA, statistical analysis was conducted on data sets of *n* = 3, with singular samples collected from 3 independent experiments. Quantitative values are expressed as mean ± SE or SD, as noted in the figure legends. The Shapiro–Wilk test was used to determine normality of data sets. If the data was normally distributed, a one-way ANOVA with Tukey’s post hoc test was used. If normality could not be confirmed, the Kruskal–Wallis test was used. Analysis was performed using the Origin(Pro) Version 2022 software (OriginLab Corporation, Northampton, MA, USA) Statistical significance was defined as * *p* < 0.05, ** *p* < 0.01, and *** *p* < 0.001. No statistical significance was defined as *p* ≥ 0.05.

## 3. Results

### 3.1. hcBMSC Sheets Respond to IFN-γ in a Dose-Dependent Manner

IFN-γ is a potent stimulator of MSC immunosuppressive properties; hence, we sought to determine how hcBMSC sheets responded to different in vitro concentrations of IFN-γ. Qrt-PCR analysis reveals that IFN-γ stimulates hcBMSC sheet expression of genes encoding immunosuppressive factors IL-10, IDO-1, PD-L1, and HLA-DR, but not PTGES2 (Supplemental Figure S2). After 48 h of culture, a peak hcBMSC sheet response was seen at 25 ng/mL of added IFN-γ in culture. These data indicate that 25 ng/mL IFN-γ may be the optimal ex vivo priming concentration to elicit maximum hcBMSC sheet immunosuppressive activity in these cultures. Notably, even at the lowest IFN-γ concentration, 0.25 ng/mL, substantial upregulation of IDO-1, PD-L1, and HLA-DR was observed (Supplemental Figure S2c,d,f).

### 3.2. hcBMSCs Readily Detach as 3D Cell Sheets after IFN-γ Priming

**Figure 1:**
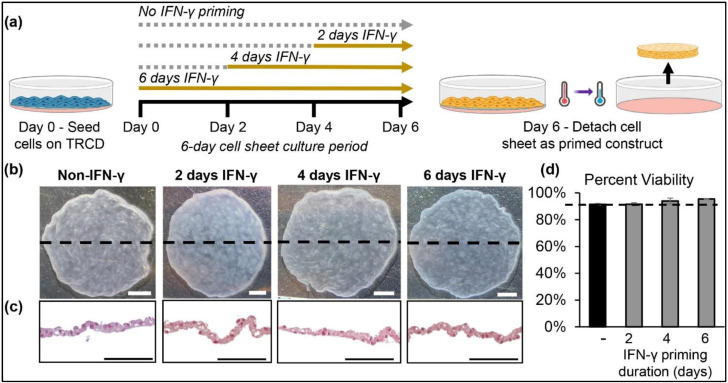
hcBMSCs readily detach as 3D sheets after treatment with IFN-γ. (**a**) Experimental schematic of fabrication of IFN-γ primed hcBMSC sheets with IFN-γ added at different timepoints of the 6-day cell sheet culture to elicit different priming durations. (**b**) Representative macroscopic images of detached 3D contracted cell sheet constructs (scale bar = 2 mm). (**c**) Representative cross-sectional hematoxylin and eosin staining of non-primed and primed cell sheet constructs (scale bar = 100 µm). (**d**) Cell viability of non-primed and primed cell sheets after temperature-mediated detachment (*n* = 2). Dotted line = 90%. Error bars represent means ± SE.

MSCs are reported to readily detach as 3D constructs following temperature-mediated release from temperature-responsive culture dishes (TRCDs) [53,54]. Here, we fabricated IFN-γ primed cell sheets by seeding hcBMSCs onto TRCDs with 25 ng/mL IFN-γ added at day 0, 2, or 4 and cultured as a 2D monolayer under high confluency conditions (Figure 1a), resulting in a contracted 3D cell sheet construct following temperature-mediated detachment (Figure 1b). Hematoxylin and Eosin (H&E) staining of IFN-γ-primed and non-primed hcBMSC sheet cross-sections shows that all hcBMSC sheets are contiguous constructs with neighboring cells adjoined (Figure 1c). The primed hcBMSC sheets’ cell viability was comparable to non-primed sheets, ≥90%, indicating no IFN-γ related toxicity in cell viability (Figure 1d). These data demonstrate that IFN-γ supplementation during cell sheet preparation does not negatively affect cell sheet fabrication and quality, indicating a viable strategy to produce IFN-γ-primed hcBMSC sheets (IFN-γ-hcBMSC).

### 3.3. IFN-γ Priming Duration Directly Relates to hcBMSC Gene Expression

**Figure 2:**
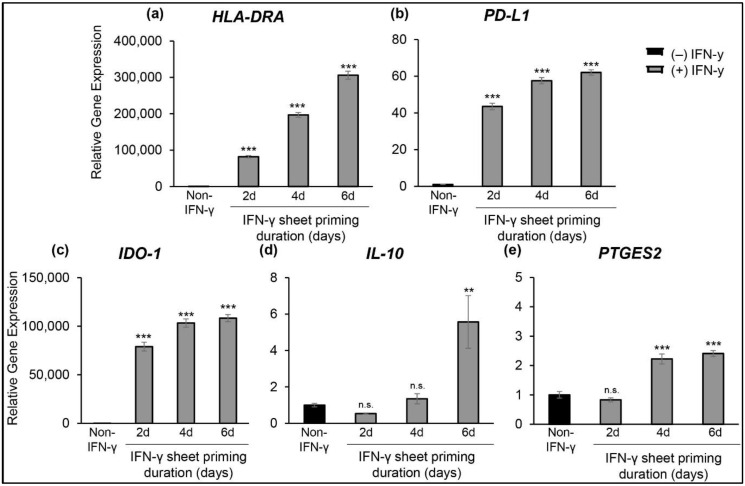
Duration of IFN-γ priming is directly related to enhanced hcBMSC sheet gene expression of immunomodulatory factors. Quantitative real-time PCR gene expression for immunomodulatory genes (**a**) human leukocyte antigen DR (HLA-DRA), (**b**) programmed death ligand-1 (PD-L1), (**c**) indoleamine 2,3-dioxygenase (IDO-1), (**d**) interleukin 10 (IL-10), and (**e**) prostaglandin E synthase 2 (PTGES2). All gene expression is normalized to GAPDH, fold change is relative to non-primed control hBMSC sheet sample. Error bars represent means ± SE (*n* = 6) (n.s. *p* ≥ 0.05, ** *p* < 0.01, *** *p* < 0.001) Statistical analysis *p* values are compared to non-IFN-γ hcBMSC sheets (non-IFN-γ, control).

qRT-PCR analysis of IFN-γ-hcBMSC sheets immediately following detachment (day 0) reveals that longer priming duration leads to upregulated expression of IL-10, IDO-1, PTGES2, PD-L1, and HLA-DR (Figure 2). After 2 days of priming, hcBMSC sheets significantly upregulate expression of HLA-DR (8.2 × 10^3^-fold change), PD-L1 (43.5-fold change), and IDO-1 (7.9 × 10^3^-fold change), but decreased IL-10 (0.5-fold change), and PTGES2 (0.8-fold change). Priming for 4 vs. 6 days similarly upregulates expression of HLA-DR (19.7 × 10^3^-fold change vs. 30.6 × 10^3^-fold change), PD-L1 (57.6-fold change vs. 62.1-fold change), IDO-1 (i.e., 10.3 × 10^3^-fold change vs. 10.8 × 10^3^-fold change, respectively), in addition to IL-10 (1.3-fold change vs. 5.6-fold change), and PTGES2 (2.2-fold change vs. 2.4-fold change) (i.e., 4-day primed vs. 6-day primed) (Figure 2d,e). These data confirm that priming with IFN-γ produces hcBMSC sheets with upregulated immunosuppressive properties in both time- and dose-dependent manners.

### 3.4. IFN-γ Primed hcBMSC Sheets Upregulate Soluble Factor Secretion for 4 Days Post-IFN-γ Removal

**Figure 3:**
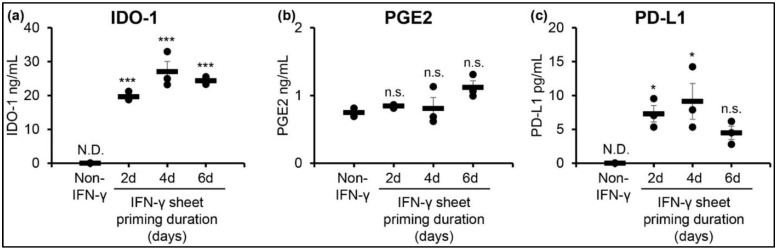
IFN-γ primed hcBMSC sheets exhibit upregulated cytokine secretion 4 days post IFN-γ removal. Enhanced levels of (**a**) IDO-1, (**b**) PGE2, and (**c**) PD-L1 secretion from IFN-γ-hcBMSC sheets was detected 4 days after removing the IFN-γ stimulation. Error bars represent means ± SE (*n* = 3, independent experiments) (n.s. *p* ≥ 0.05, * *p* < 0.05, *** *p* < 0.001) Statistical analysis *p* values are compared to non-IFN-γ hcBMSC sheets (non-IFN-γ, control).

To understand the enduring effects of IFN-γ priming on hcBMSC sheets, we harvested primed and non-primed hcBMSC sheets and re-plated them onto cell cultureware in media without IFN-γ supplementation. After 4 days, we measured the secretion of soluble factors IL-10, IDO-1, PGE2, and PD-L1 in the media. All IFN-γ-hcBMSC sheets produced significantly increased secretion of IDO-1, ranging from 19.6–27.0 ng/mL, compared to non-IFN-γ hcBMSC sheets, which had non-detectable levels of IDO-1 (Figure 3a). Similarly, IFN-γ-hcBMSC sheets exhibit greater production of PD-L1 (4.5–13.9 pg/mL) and PGE2 (0.78–1.1 ng/mL) compared to non-primed hcBMSC sheets (Figure 3b,c). The clear priming duration affect observed in gene expression at day 0 was not present in protein secretion levels by day 4 post-IFN-γ priming. Between primed cell sheets, the highest IDO-1 secretion was observed in the 4-day primed IFN-γ-hcBMSC sheet, the highest PGE2 in the 6-day primed IFN-γ-hcBMSC sheet, and highest PD-L1 in the 4-day IFN-γ-hcBMSC sheet. IL-10 secretion levels of all samples were below the limit of detection (≤31.2 pg/mL). These data demonstrate that IFN-γ has a lasting effect on hcBMSC sheets after the removal from cell culture medium for at least up to four days, evidenced by persistent in vitro upregulation of immunomodulatory molecules IDO-1, PGE2, and PD-L1 after removal of IFN-γ.

### 3.5. IFN-γ Primed hcBMSC Sheets Inhibit T Cell Proliferation in Indirect Coculture

**Figure 4:**
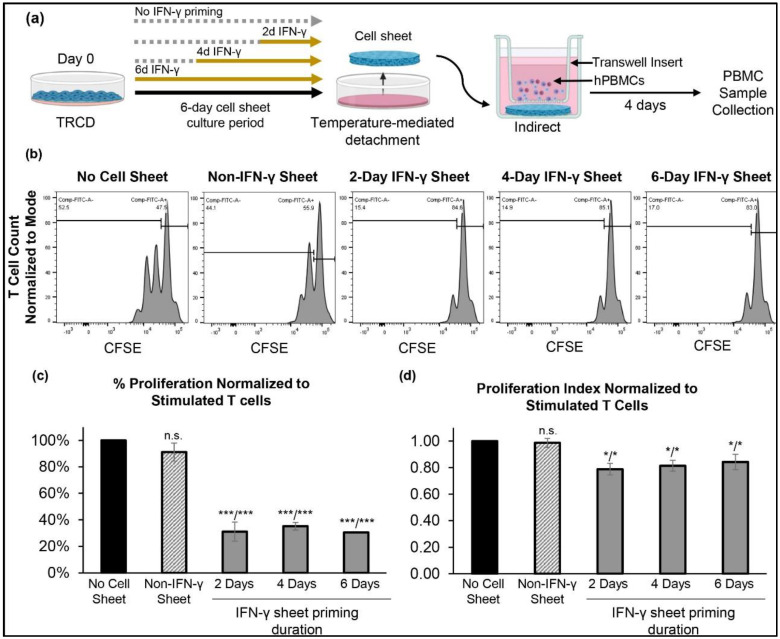
IFN-γ primed hcBMSC sheets exhibit enhanced immunosuppressive activity in indirect coculture with hPBMCs. (**a**) Experimental schematic of hcBMSC sheet fabrication and T cell proliferation coculture assay. (**b**) Representative flow cytometry plots of gated T cells −/+ hcBMSC sheet indirect coculture. Quantification of (**c**) percent proliferated T cells (divided cells) and (**d**) proliferation index normalized to stimulated T cells in PBMC control, no coculture, group. Error bars represent means ± SD (*n* = 3, independent experiments) (n.s. *p* ≥ 0.05, * *p* < 0.05, *** *p* < 0.001). Statistical analysis *p* values are represented as compared to no cell sheet/non-IFN-γ sheet.

To assess the immunosuppressive activity of primed and non-primed hcBMSCs as transferable cell sheet constructs, detached hcBMSC sheets were subjected to indirect transwell coculture with isolated hPBMCs (Figure 4a) [52,55]. Indirect coculture enables evaluation of secretome-mediated paracrine signaling. We observed that IFN-γ-hcBMSC sheets in indirect coculture with hPBMCs, significantly decreased proliferation of stimulated T cells compared to non-IFN-γ hcBMSC sheets and hPBMCs cultured in the absence of cell sheet coculture (positive control) (Figure 4b–d). All IFN-γ-hcBMSC sheets significantly decreased the proliferation of T cells by 31–35% (Figure 4c). Non-primed hcBMSC sheets did not significantly affect T cell proliferation and only minimally decreased their proliferation by 91% (Figure 4c). Indirect coculture with IFN-γ-hcBMSC sheets also resulted in a significantly decreased normalized proliferation index by around 80%, while non-IFN-γ hcBMSC sheets, had no effect on the proliferation index (Figure 4d). No significant differences were observed in immunosuppressive activity between IFN-γ-hcBMSC sheets primed for different durations, suggesting that 2 days of priming prior to cell sheet detachment is sufficient for fabricating highly immunomodulatory MSC sheets. Together these data indicate that IFN-γ priming is a feasible strategy to significantly enhance the immunosuppressive secretory paracrine activities of hcBMSC sheets.

### 3.6. Cell-Contact Increases Immunosuppression Activity of Primed and Non-Primed hcBMSC Sheets

**Figure 5:**
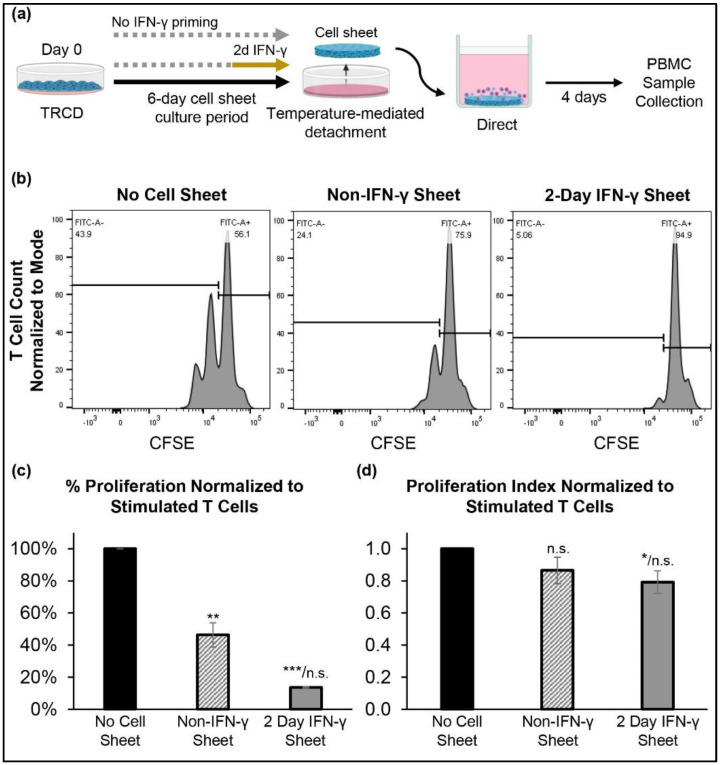
Non-IFN-γ and IFN-γ primed hBMSC sheets exhibit enhanced immunosuppressive activity in direct coculture with hPBMCs. (**a**) Experimental schematic of hcBMSC sheet fabrication and T cell proliferation coculture assay. (**b**) Representative flow cytometry plots of gated T cells −/+ hcBMSC sheet indirect coculture. Quantification of (**c**) percent proliferated T cells (divided cells) and (**d**) proliferation index normalized to stimulated T cells in PBMC control, no coculture, group. Error bars represent means ± SD (*n* = 3, independent experiments) (n.s. *p* ≥ 0.05, * *p* < 0.05, ** *p* < 0.01, *** *p* < 0.001). Statistical analysis *p* values are represented as compared to no cell sheet/non-IFN-γ sheet.

To further assess the immunosuppressive abilities of hcBMSC sheets, we performed direct cocultures to evaluate both the effects of secretome-mediated paracrine signaling and direct cell contact between hPBMCs and hcBMSC sheets (Figure 5a). Given the similar immunosuppressive activity of the different IFN-γ-hcBMSC sheets in indirect coculture, indicated by T-cell proliferation inhibition (Figure 4b–d), here, we chose to only investigate the 2-day IFN-γ-hcBMSC sheet condition. When directly cocultured with hPBMCs, the IFN-γ-hcBMSC sheets and non-IFN-γ hcBMSC sheets both significantly decreased the percent of proliferated activated T cells by 14 ± 1% and 46 ± 10%, respectively, compared to hPBMCs cultured in the absence of cell sheet coculture (Figure 5b,c). The IFN-γ-hcBMSC sheet also significantly inhibited T cell proliferation compared to the non-primed hcBMSC sheets (Figure 5c). IFN-γ-hcBMSC sheets, but not non-primed hcBMSC sheets, significantly decreased the proliferation index of activated T cells (Figure 5d), similar to indirect coculture. Furthermore, when we compared the effect of indirect and direct coculture, we observed significantly enhanced immunosuppressive activity in the direct cocultures, measured by inhibited T cell proliferation, in both non-primed and IFN-γ-hcBMSC sheet groups (comparison not shown). These results suggest that IFN-γ priming significantly enhances both direct and indirect suppression of T cells, and that the combination of direct contact and indirect paracrine signaling pathways are essential for maximal hcBMSC sheet-mediated immunosuppression.

### 3.7. Concentration of Soluble Immunosuppressive Factors Increases in hcBMSC Sheet-hPBMC Coculture

**Figure 6:**
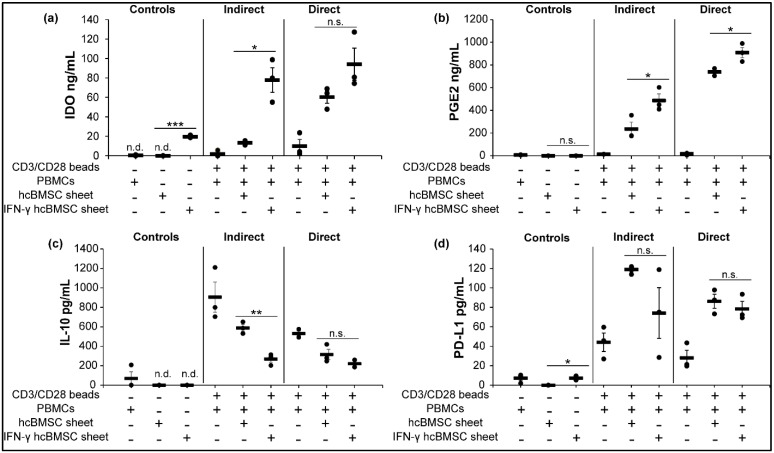
hcBMSC sheet PBMC coculture stimulates production of immunosuppressive factors. Enhanced (**a**) IDO-1, (**b**) PGE2, levels relate to enhanced immunosuppression in hcBMSC sheet coculture groups. (**c**) Decreased IL-10 levels relate to enhanced immunosuppression in hcBMSC sheet coculture groups. (**d**) PD-L1 was upregulated in all cell sheet PBMC coculture groups unrelated to observed immunosuppression. Error bars represent means ± SE (*n* = 3, independent experiments) (n.s. *p* ≥ 0.05, * *p* < 0.05, ** *p* < 0.01, *** *p* < 0.001).

To better understand the relationship between levels of anti-inflammatory soluble immunosuppressive factors and observed immunosuppression in hcBMSC sheet hPBMC cocultures, we measured the protein levels of IFN-γ induced immunomodulatory molecules (IL-10, IDO-1, PGE2, PD-L1) within the hcBMSC sheet hPBMC cocultures using ELISA. Both IDO-1 and PGE2 secretion levels related positively to observed increased inhibition of T cell proliferation, with highest concentrations observed in direct coculture, where the highest immunosuppression of T cells was observed (Figure 6a,b). IFN-γ-hcBMSC sheet-hPBMC coculture resulted in significantly higher IDO-1 (Figure 6a) and PGE2 (Figure 6b) in both indirect and direct coculture. Conversely, IL-10 secretion levels (Figure 6c) were inversely related to the upregulated immunosuppressive activity of hcBMSC sheets, determined by inhibited T cell proliferation. IFN-γ-hcBMSC sheet-hPBMC coculture produced the lowest secretion levels of IL-10. PD-L1 levels (Figure 6d) increased in all cocultures, unrelated to IFN-γ priming or immunosuppressive trends. These data indicate that IFN-γ priming leads to enhanced IDO-1 and PGE2 production in coculture, related to immunosuppression of T cells, and that hcBMSC sheet hPBMC coculture produces a microenvironment with upregulated anti-inflammatory factors IDO-1, PGE2, and PD-L1.

## 4. Discussion

IFN-γ priming of MSCs has been extensively reported to improve MSC-mediated immunosuppression by upregulating expression of paracrine factors [27,31,32]. Clinical translation, however, remains hindered by the current standard practices utilizing suboptimal injection-based delivery of MSC suspensions after priming, limiting tissue site engraftment and the therapeutic paracrine activity window [7]. To address this limitation, we exploit known cell sheet technology to fabricate transplantable, scaffold-free constructs capable of superior MSC retention and localization [37,38,39,40]. The enhanced MSC sheet engraftment provides increased opportunity for immune interactions compared to rapidly cleared injected or infused MSC suspensions. Although, the immunosuppressive ability of allogeneic MSCs as cell sheets remains currently underreported. Therefore, a better understanding and enhancement of the immunosuppressive activity of MSC sheets is required to establish an immunosuppressive hcBMSC sheet for future clinical use.

This study aims to determine the immunosuppressive potential of hcBMSCs [42] as cell sheets and employ an IFN-γ priming method to enhance their immunosuppressive activity, measured using an in vitro human hPBMC coculture assay. The hcBMSCs used in this study demonstrate clinical safety as an allogeneic infused cell therapy product, contributing to the future translatability of the results presented here [42]. Our central finding was that IFN-γ priming significantly enhanced hcBMSC sheet contact-independent and contact-dependent immunosuppressive activity by upregulating production of anti-inflammatory paracrine factors IDO-1, PGE2, and PD-L1.

This study reports the first successful fabrication of IFN-γ-primed hcBMSCs as transferable 3D cell sheets (Figure 1). hcBMSC sheets at time of delivery are considered to be a 3D structure (Figure 1c), that is significantly thicker than hBMSCs in adherent monolayer culture, as previously shown [57]. At baseline, non-primed hcBMSC sheets express low levels of immunosuppressive paracrine factors, IDO-1, IL-10, PD-L1, PGE2, and HLA-DR, which are significantly upregulated in response to IFN-γ priming (Figure 2). Following previous reports [58], our data indicated that priming duration positively correlates to upregulated hcBMSC expression of IDO-1, IL-10, PTGES2, PD-L1, and HLA-DR at day 0 (Figure 2). Furthermore, IFN-γ priming had a lasting effect on hcBMSC sheets, evidenced by persistent upregulation of secreted soluble factors IDO-1, PGE2, and PD-L1 four days after removal of IFN-γ from culture (Figure 3). IL-10 was below the limit of detection (≤31.2 pg/mL). Four days after IFN-γ removal, IFN-γ priming duration, from 2 to 6 days, no longer directly related to upregulated expression, revealing that shorter dose durations are equally capable of retaining a lasting priming effect. Towards future clinical translation, this lasting priming effect implicates that post transplantation the IFN-γ-hcBMSC sheets will retain upregulated production of immunosuppressive factors.

MSCs as a 2D adherent monolayer (standard culture method) are well-recognized to suppress activated T cell proliferation and that this effect requires stimulation by pro-inflammatory factors [17,27,31,32]. However, adherent monolayers of MSCs on plastic do not represent the MSC delivery state in vivo. Therefore, we sought to determine the immunosuppressive activity of hcBMSCs as 3D cell sheets equivalent to their delivery state. In indirect coculture, the 2-day, 4-day, and 6-day IFN-γ-primed hcBMSC sheets are all capable of significantly inhibiting stimulated T cell proliferation compared to non-primed hcBMSC sheets, measured by the percent of proliferated T cells and the average number of divisions in divided cells (proliferation index) (Figure 4b–d). Thus IFN-γ priming successfully upregulated paracrine signaling mediated immunosuppressive paracrine activity of hcBMSC sheets, inhibiting T cell proliferation without cell contact. No significant differences were observed between the different priming durations (Figure 4b–d), supporting that shorter IFN-γ priming durations successfully boost hcBMSC sheet immunosuppressive activity. This finding is further supported by previous studies reporting higher MSC immunosuppressive potency following shorter priming durations compared to longer priming durations [58]. In direct coculture, we also demonstrated that IFN-γ-hcBMSC sheets significantly reduced the percent of T cell proliferation and proliferation index compared to hPBMCs cultured independently (Figure 5b). Notably, direct coculture significantly augmented both the non-primed and IFN-γ-hcBMSC sheets ability of inhibit T cell proliferation, reducing the fraction of divided cells (Figure 5b) compared to indirect coculture (Figure 4b), consistent with previously reported studies of MSCs in 2D monolayers [59,60]. Only the IFN-γ-hcBMSC sheets showed the ability to significantly reduce both the percent of proliferated T cells and proliferation index in both indirect (Figure 4c,d) and direct coculture (Figure 5c,d). Taken together, IFN-γ priming enhanced hcBMSC sheet contact-independent and -dependent immunosuppression, with maximal immunosuppression during direct cell coculture, where cell–cell contact, and secretory paracrine pathways could work in combination [13]. This direct cell–cell contact effect between the cell sheet and PBMCs suggests that the long-term localized engraftment of transplanted MSC sheets at the target tissue site may be critical to boosting therapeutic immunosuppressive affects via increased opportunity for direct cell contact.

These findings are further supported by results demonstrating that direct cell-to-cell interactions between MSCs and hPBMCs facilitate greater bidirectional communication yielding enhanced immunosuppressive microenvironment [13,18,59,61]. Direct cell contact between MSCs and hPBMCs is reported to facilitate mitochondrial transfer via HLA binding pathways, reportedly upregulating the immunoregulation of T cells [61]. Specifically, allogeneic HLA-C and HLA-DRB1 mismatch positively correlated with higher ability of MSCs to promote and support Treg cells [61]. HLA-G, a molecule involved in fetal tolerance, is also shown to promote MSC suppression of T cells and NK cells, and promote Treg cells [21]. These direct communication pathways upregulate bidirectional cross-talk between stimulated PBMCs and MSCs to enhance production of immunosuppressive factors including IDO-1 and CXCL10 [18].

In addition to direct cell–cell contact interaction, IFN-γ priming of hcBMSC sheets prior to coculture enhanced production of IDO-1 and PGE2 via paracrine signaling during coculture (Figure 6). Follow-up ELISA analysis of soluble immunomodulatory molecule secretion during coculture showed an overall shift in IDO-1 and PGE2 production between non-IFN-γ and IFN-γ primed hcBMSC sheets related to the immunosuppressive activity (Figure 6). IDO-1 and PGE2 are reported to have non-redundant, essential roles in modulating allo-immunosuppression [62]. IDO-1 is a potent IFN-γ inducible enzyme that catalyzes the first step in the tryptophan to kynurenine pathway, inducing tryptophan depletion in T cells leading to inhibited proliferation and stimulation of Treg cells [16,17,58,63]. PGE2 suppresses T cell proliferation through binding EP2 and EP4 receptors, inhibiting T cell activation [13,59]. The concentration of IDO-1 and PGE2 in IFN-γ-hcBMSC sheet hPBMC coculture directly related to inhibition of T cell proliferation, implicating a positive relationship between increased IDO-1 and PGE2 and hcBMSC sheet immunosuppressive activity, consistent with prior reports [17,22].

Surprisingly, PD-L1 levels were comparably upregulated in both the IFN-γ-hcBMSC sheet and non-primed hcBMSC sheet coculture groups (Figure 6d). MSC expression of PD-L1 either as a transmembrane ligand or as a secreted factor is reported to inhibit T cell activation via the PD-L1/PD-1 pathway [19,64]. However, the direct role of PD-L1 in MSC-mediated immunosuppression is conflicting. English et al. [62] reported that PD-L1 is not essential to MSC immunomodulatory effects, while Davies et al. [19] showed that anti-PD-L1 treatment reduced MSC-mediated T cell inhibition. Here, we observed the highest soluble PD-L1 concentrations in non-primed hcBMSC sheet indirect coculture, where the lowest immunosuppressive activity was seen (Figure 4), suggesting that soluble PD-L1 does not contribute to inhibition of T cells, at least in indirect pathways. Instead, PD-L1 as a transmembrane protein may play a more critical role in direct coculture where increased immunosuppressive activity was observed. Further evaluation of cell surface expression of PD-L1 would confirm these effects. Nonetheless, it is intriguing that of the factors tested, the non-primed sheets upregulated PD-L1 the most in indirect coculture.

In contrast, IL-10 levels were inversely related to the inhibition of T cell proliferation (Figure 6c). hcBMSC sheet coculture decreased IL-10 in relation to inhibited T cell proliferation (Figure 4 and Figure 5). The role of IL-10 in MSC-mediated immunosuppression is debated as it is often barely detectable in MSCs as we observed in Figure 6, and literature surrounding the trends of IL-10 secretion and hPBMC proliferation is conflicting [12,22]. Additionally, treatment with anti-IL-10 is not reported to reduce MSC abilities to inhibit T cell proliferation [17]. In this study, IFN-γ-hcBMSC sheet coculture exhibited the lowest IL-10 concentration while simultaneously producing the highest immunosuppressive effects, suggesting that IL-10 did not contribute to the observed immunosuppression of T cell proliferation observed in this assay.

As expected with IFN-γ priming, hcBMSC sheets highly express MHC II molecules (e.g., HLA-DR, Figure 2) in addition to innately expressed MHC I [65]. Our data demonstrate that this does not impede MSC sheet allo-immunosuppression in direct coculture (Figure 5). We also observed no proliferation of non-stimulated T cells when directly cocultured with IFN-γ-hcBMSC sheets (data not shown). However, the induction of memory and plasma cells remains an important area for further investigation. These experiments would be critical for understanding the potential long-term immunogenicity of allogeneic MSC sheets post-transplantation in vivo. Interestingly, a recent phase II GvHD study using MSCs reported that response rate to MSC treatment was unrelated to HLA matching [66] and that, as previously mentioned, the immunosuppressive potential of MSCs may be positively related to allogeneic HLA-C and HLA-DRB1 mismatch load [61]. Accordingly, an equine osteoarthritis model reported decreased white blood cell (WBC) counts in allogeneic primed BMSCs compared to non-primed BMSCs following the first administration, but did show elevated WBC counts compared to non-primed BMSCs following a second repeat administration, however this did not relate to decreased therapeutic effect [67].

In vitro functional assays of MSCs described here are essential to identifying allogeneic human MSC immunosuppressive activities and mechanisms challenging to assess in xenogeneic animal implant models due to species variabilities [68]. While this in vitro study provides valuable insight into the immunosuppressive potential of MSC sheets, limitations to the current study must be considered. Here, immunosuppression was defined as inhibiting T cell proliferation, an indicator of reduced T cell activation, and did not consider additional markers of T cell activation/phenotype, such as IL-2 and granzymes, or functional changes in other cell types such as macrophage polarization. Furthermore, while we focused on thoroughly reported MSC-produced immunomodulatory molecules IL-10, IDO-1, PGE2, and PD-L1, we acknowledge that other IFN-γ-induced chemokines, cytokines, and growth factors likely play a role here as well [18]. These studies would improve mechanistic understanding of the effects observed within hPBMC cocultures. In addition to the use of PBMCs, coculture with individually isolated T cells and other immune subsets would provide deeper insight into the cell-specific mechanistic interactions and immunosuppressive pathways. Future allogeneic in vivo animal transplantation models will also be critical to understanding therapeutic potential and establishing IFN-γ-hcBMSC sheets as a promising treatment to reduce inflammation in immune-related diseases. MSC sheet-induced T cell modulation could be elicited in local disease site sheet placement via MSC autocrine and paracrine signaling, and from remote cell sheet placement by MSC sheet paracrine signals.

In summary, this study demonstrates that IFN-γ priming during hcBMSC sheet fabrication is a feasible strategy towards generating immunomodulatory transferable MSC constructs, and that IFN-γ significantly upregulates hcBMSC sheet contact-independent and -dependent immunosuppression by upregulating immunosuppressive factors IDO-1, PGE2, and PD-L1. Based on these findings, we assert the combination of IFN-γ priming and MSC sheets as a platform to design highly immunosuppressive hcBMSC sheets to improve MSC-mediated therapy for immune-related disorders.

## Data Availability

Data sets generated in this study are available from the corresponding author upon reasonable request.

## Supplementary Materials

The following supporting information can be downloaded at: https://www.mdpi.com/article/10.3390/cells11233738/s1, Figure S1: hPBMC gating strategy; Figure S2: hcBMSC sheets respond to IFN-γ in a dose-dependent manner.
