# Supplementary material for: Interferon-Gamma Primed Human Clonal Mesenchymal Stromal Cell Sheets Exhibit Enhanced Immunosuppressive Function
Source: Cells. 2022 Nov 23;11(23):3738. doi: 10.3390/cells11233738 (PMC9737548; doi:10.3390/cells11233738)
Supplement: Supplementary file 1 [file cells-11-03738-s001.zip › cells-2017889-supplementary.pdf]

Supplemental Figures

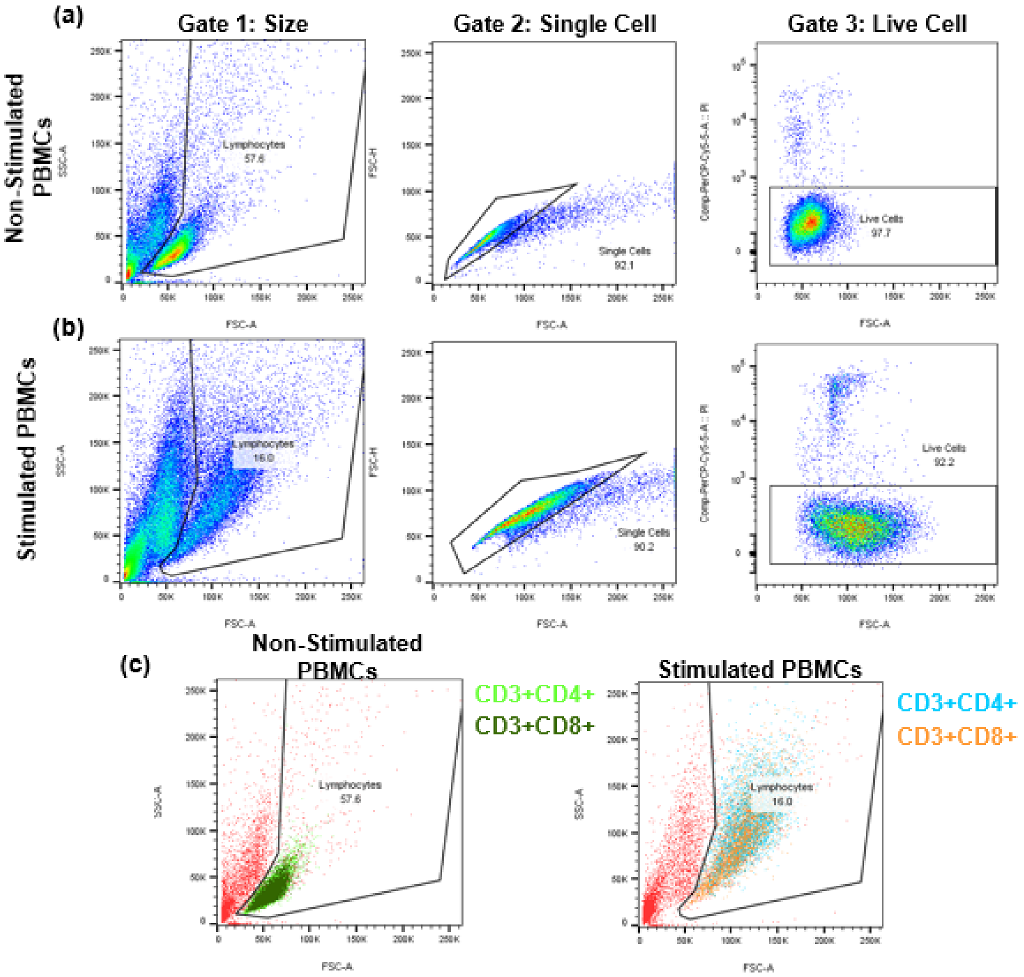

Figure S1. hPBMC gating strategy. Prior to measuring CSFE stained T cell populations, (**a**) non-stimulated and (**b**) stimulated T cells within PBMCs were gated by size (FSC-A vs. SSC-A), single cell (FSC-A vs. FSC-H), and live/dead cells (PI). (**c**) This gating strategy resulted in a population of cells that were CD3+CD4+ and CD3+CD8+.

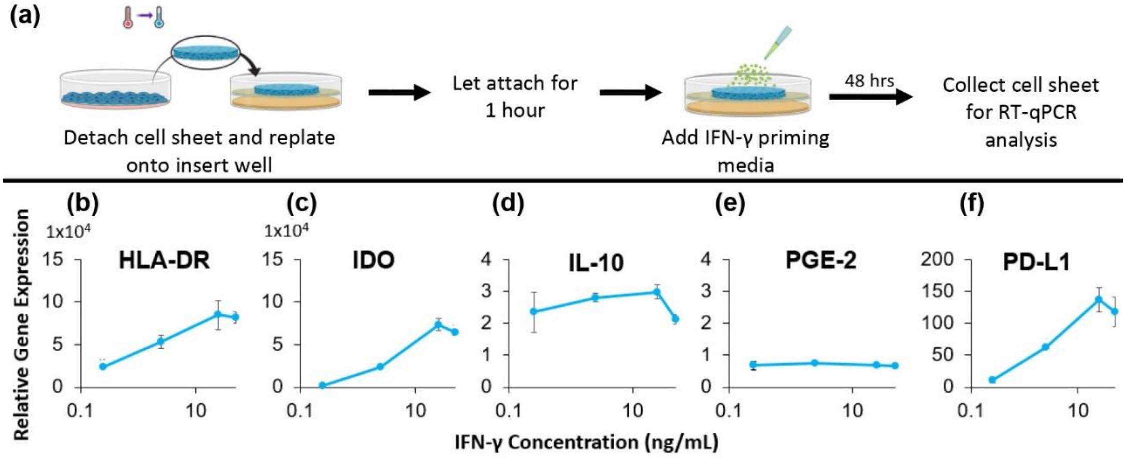

Figure S2. hcBMSC sheets respond to IFN-γ in a dose-dependent manner. (**a**) Experimental schematic. Quantitative real-time PCR gene expression for immunomodulatory genes (**b**) human leukocyte antigen DR (HLA-DR), (**c**) indoleamine 2,3-dioxygenase (IDO-1), (**d**) interleukin 10 (IL-10), and (**e**) prostaglandin E synthase 2 (PTGES2), and (**f**) programmed death ligand-1 (PD-L1). All gene expression normalized to GAPDH and compared to non-primed control hBMSC sheet sample. Error bars represent means ± SD (n = 2).
